# Vertical Tropia Following Horizontal Transposition Surgery

**DOI:** 10.22599/bioj.160

**Published:** 2021-03-12

**Authors:** A. Thomas, P. Watts

**Affiliations:** 1 University Hospital of Wales, GB

**Keywords:** vertical tropia, horizontal transposition surgery, Duane syndrome, sixth nerve palsy

## Abstract

**Aim:**

The aim of this study was to determine the prevalence of vertical tropia following horizontal transposition of both vertical rectus muscles (HToVR) in patients with Duane syndrome or sixth nerve palsy.

**Methods:**

This retrospective study included patients with Duane syndrome or sixth nerve palsy who had undergone HToVR muscles. Data collected included: age, gender, diagnosis, laterality, pre-operative angle of deviation, type of surgery and post-operative angle of deviation at one week, three months and six months. Information on the use of botulinum toxin (BT) ipsilateral medial rectus (MR), additional surgery was performed, and the presence of preoperative and postoperative binocular function and any vertical deviation was collected.

**Results:**

There were 11 patients, eight patients with a diagnosis of Duane syndrome and three patients with a diagnosis of sixth nerve palsy. The mean age of the patients was 13 ± 14.79 years (range 5–55 years), four were female. The prevalence of post-operative vertical tropia was 54%. The mean vertical deviation for distance, was 7.6^ ± 2.94 (SD) (range 3^–9^). Stereoacuity was present preoperatively in 5 patients and 8 postoperatively. No patient developed diplopia or received further surgery for the vertical tropia. Of the six patients who had intraoperative BT at the time of the HToVR, four developed a vertical deviation.

**Conclusion:**

The prevalence of vertical deviation following HToVR muscles was 54% in our series. None of the patients with an induced postoperative vertical deviation reported diplopia or required further surgery for it.

## Introduction

Horizontal transposition of the vertical rectus (HToVR) muscles refers to transposing whole or part of the muscle in order to change its primary or secondary action. It has been used to treat type 1 Duane syndrome or unrecovered sixth nerve palsies (Ansons and Davis 2001).

The HToVR has been shown not only to correct the esotropia associated with Duane syndrome or sixth nerve palsies but also increase the amount of abduction (Ansons and Davis 2001). Modification of the procedure with the placement of posterior fixation sutures has been reported to increase the amount of horizontal deviation corrected and improve the abduction forces (Foster 1997).

However, it has been shown that there is a risk of inducing a vertical tropia following transposition surgery (Ruth, Velez and Rosenbaum 2009) (Dagi and Elhusseiny 2020). Therefore, the aim of this study was to determine the prevalence of vertical tropia following horizontal transposition of the vertical rectus muscles (HToVR) in patients with Duane syndrome or sixth nerve palsy.

## Methods

Data were gathered from the notes of patients that had a diagnosis of type 1 Duane syndrome or unrecovered sixth nerve palsy, undergoing surgical intervention with HToVR muscles, between June 2003 and March 2012. The details of the surgical procedure have been previously described (Schillinger 1959). All patients had an identical surgical procedure by the same surgeon (PW); the vertical recti muscles were transposed to the lateral rectus along the spiral of Tillaux augmented with a Foster suture (Foster 1997). This suture improves the abducting vector forces of the transposed vertical recti. This involves two sutures: one for the transposed superior rectus (SR) and one for the transposed inferior rectus (IR). The suture includes a bite of inferior border of the SR, 7mm from the lateral rectus (LR) insertion and sutured to the sclera just above the LR superior border. Similarly, another suture includes a bite of the superior border of the transposed inferior rectus 7mm from the LR insertion and sutured to the sclera at the lower border of the LR. If botulinum toxin (BT) was used, it was injected via a trans–conjunctival route into the medial rectus at the time of the surgical procedure. BT is given to the medial rectus (MR) to reduce the pull of the MR while the transposed muscles heal into position.

Data collected included age at time of surgery, gender, diagnosis, laterality, preoperative angle of deviation (near and distance), type of surgery, postoperative angle of deviation for (near and distance) at one week, three months and six months follow up post–operatively, preoperative and postoperative binocular vision and additional surgery postoperatively. All measurements were done using the prism cover test. Ethical approval was sought but deemed not necessary. The study complied with the principles of the Declaration of Helsinki.

## Results

There were 11 patients, with seven males and a mean age of 13 ± 14.79 years (range 5–55 years). There were eight adults and three children.

The prevalence of postoperative vertical deviation in this case series was 54% (6/11 patients) at the last follow up; where four patients had ipsilateral hypertropia, one patient had contralateral hyperphoria and one had ipsilateral hypotropia. Of the eight patients with Duane syndrome, five patients (Patients 5, 6, 7, 9 and 11 in ***Table 1***) had a vertical deviation (62%). In the group with the sixth nerve palsy, Patient 1 (***Table 1***) already had an ipsilateral hypertropia prior to the transposition surgery.

**Table 1:** A table showing the preoperative and postoperative results at six months of all patients included in the study.(*post–operative measurements at 3 months due to patient being discharged at this point).


PATIENT	AGE	DIAGNOSIS	PRE OP D/N	POST OP D/N	VERTICAL PRE OP D/N	VERTICAL POST OP D/N	ADDITIONAL PROCEDURE	PRE–OP BV	POST–OP BV	LENGTH OF FOLLOW UP (MONTHS)

1*	24	Sixth N palsy	30^RET/25^RET	Nil	8^RHT/12^RHT	3^RHT/6^RHT	Nil	Nil	Nil	3

2	11	Sixth N palsy	40^LET/35^LET	14^LET/20^LET	nil	Nil	BT to LMR & then MR recession	Nil	Nil (after LMR recession 100” of arc)	6

3	55	Sixth N palsy	50^/RET/35^RET	6^RX(T)/6^RH(T)	4^RHT/nil	12^RH(T)/4^RH(T)	Nil	Nil	200” of arc at last f/u	6

4	7	Duane	12^LET/12^LET	6^X/6^X	nil	Nil	Nil	200” of arc c AHP	100” of arc at last f/u	33

5	5	Duane	40^RET/30^RET	8^LET/10^LET	nil	8^LHT/nil	Nil	Nil	400” of arc at last f/u	20

6	8	Duane	20^LET/12^LET	Nil	nil	10^LH/9^LH	Nil	100” of arc c AHP	40” of arc at last f/u	13

7	5	Duane	12^LET/10^LET	8^LET/8^LET	nil	Nil	Nil	80” of arc c AHP	40” of arc c AHP	12

8	2	Duane	unknown/35^LET	16^RET/E	nil	Nil	Nil	Nil	140” of arc at last f/u	58

9	22	Duane	18^LET/18^LET	25^LET/35^LET	nil	2^LHT/3^LHT	BT to MR	40” of arc c AHP	40” of arc c AHP	8

10	9	Duane	18^LET/10^LET	25^LET/20^LET	nil	Nil	BT to MR & then LMR recession	200” of arc c AHP	Nil	14

11	5	Duane	30^RET/35^RET	18^L/AET/30^L/AET	nil	Nil	Nil	Nil	Nil	53

11	6	Duane	18^L/AET/30^L/AET	14^LET/18^LET	nil	6^LHyopT/8^LhypoT	Nil	Nil	Nil	53


The median vertical deviation in these patients was 6^ (range 3^–9^) for near fixation and 7^ (range 2^–12^) for distance fixation.

No patient reported diplopia following transposition surgery. Those patients who developed a vertical deviation; one patient had a hyperphoria and were binocular (Patient 6), one was non-binocular (Patient 11) and the remaining patients (Patient 1, 3, 5 & 9) had a compensatory head posture. The compensatory head postures were reduced in most patients post–operatively. The degree of head posture was not measured pre-operatively and post-operatively; only an observation by the clinician and patient was recorded in the clinical notes.

Eight patients (72%) achieved BSV (determined by the presence of stereoacuity at their last follow up appointment (range of follow up time: 12–58 months after transposition surgery), with a median of 100 seconds of arc (ranging from 40–400 seconds of arc). We found that three patients achieved BSV following HToVR. There were five patients who had BSV with a compensatory head posture prior to transposition surgery, and four of these patients retained BSV. Following further surgery (Left MR recession) for the residual esotropia, Patient 2 (see table) regained BSV.

Patients 1, 3, 5, 6, 8 and 9 also received intra-operative botulinum toxin during the surgery. One patient required further treatment, where they received further BT and subsequently lost to follow up. There were four patients (Patients 3, 5, 6, and 9), who received intraoperative BT who developed an induced vertical deviation, compared with only one patient in the cohort who didn’t receive BT intraoperatively. None of these four patients required further treatment to correct the vertical deviation, only two patients (Patients 3 and 9) required treatment for the residual esotropia with further BT. The indication for botulinum toxin was to enhance the transposition effect during the early postoperative period.

In those patients who did not receive intraoperative BT (45%, 5/11 patients), three patients required further surgical treatment for the residual ET. This suggests that the addition of BT intra–operatively seems to show better outcomes, due to the lower risk of reoperation, for the residual ET, in this group. One patient received HToVR muscles on the other eye as the patient had bilateral Duane syndrome (patient 11).

The re-operation rate was low following HToVR. Patients 2 and 10 had further adjustable recession of their medial rectus muscles on the same side as the transposition, for residual esotropia, which was done after an interval of six months to reduce the risk of anterior segment ischaemia as decided by the surgeon (PW).

Patients 2, 9 and 10 required postoperative BT (2.5 and 5.0 units); one adult and two children.

Prior to HToVR, there were four patients (Patients 1, 3, 8 and 11) who received BT injectionsl and Patient 7 who had a MR recession. Patients 1 and 8 who had a previous BT went onto receive another injection of BT intraoperatively.

Patient 3 developed a consecutive exotropia following HToVR. However, this patient did not require further surgery. At the last follow up period of six months, the patient had an intermittent exotropia only measuring 6^ for near and distance fixation. Patient 4 developed a small exophoria measuring 6^ and 5^ for distance and near fixation respectively.

## Discussion

The prevalence of postoperative vertical deviation in both groups of patients was 54% at the last follow up; where 62% (5/8) of patients with Duane syndrome developed a vertical deviation (3/8 patients had hypertropia, one had hyperphoria and one had hypotropia). In patients with a sixth nerve palsy 33% (1/3) of patients had residual hypertropia. Elsewhere, 32% (Leiba et al. 2010) and 11% (Mehendale et al. 2012) of patients developed a vertical deviation. However, our study only had 11 patients in total compared to 22 (Leiba, Wirth, Amstutz and Landau 2010) and 17 (Mehendale, Dagi, Wu, Ledoux, Johnnston and Hunter 2012) patients. This high rate of vertical deviation has also been reported more recently by Dagi and Elhusseiny (Dagi and Elhusseiny 2020) where 50% (4/8 patients) of their cohort developed a vertical deviation. Thirty-seven and a half percent of patients with a sixth nerve palsy (3/8) developed a vertical deviation and 12.5% (1/8) with Duane syndrome developed a vertical deviation following adjustable graded augmentation of superior rectus transposition with or without medial rectus recession (Dagi and Elhusseiny 2020).

The median vertical deviation in these patients was 6^ (range 3^–9^) for near fixation and 7^ (range 2^–12^) for distance fixation.

According to (Leiba, Wirth, Amstutz and Landau 2010) the mean reduction in esotropic deviation was 30^ ± 15.8^ (range 6^–78^), compared with our study where the mean reduction was 11^ and 6^ for distance and near fixation, respectively. Elsewhere, a study demonstrated a reduction of 34^ in the angle of the esotropia (Mehendale, Dagi, Wu, Ledoux, Johnnston and Hunter 2012). They performed SR muscle was transposition along with a MR recession on adjustables. They augmented the SR muscle by placing a suture 8–12mm from the SR muscle insertion. However, (Leiba, Wirth, Amstutz and Landau 2010) opted for the more traditional option, where they performed a full tendon HToVR along with intra–operat BT injection to the ipsilateral MR, hence supporting our surgical technique in our case series.

It would seem that by transposing only the SR muscle, there has been an even greater effect on the reduction in the mean esotropia. This could be due to the use of augmenting the transposition by also recessing the MR during the same procedure, as supported by Johnston and Crouch (Johnston, Crouch and Crouch 2006) and Dagi and Elhusseiny (Dagi and Elhusseiny 2020).

Interestingly, no patient in our cohort reported vertical diplopia following their HToVR. Most patients had a residual compensatory head posture to account for their vertical deviation and one patient was not binocular. Similarly, Dagi and Elhuisseiny (2020) describe that their cohort of patients used a compensatory head posture; however, their patients did report vertical and torsional diplopia.

The limitations in our study are firstly the small number of patients we managed to gather data from, hence the higher incidence of the vertical deviations. Secondly, the retrospective nature of this study which impacts the amount of information gathered from these patients at the time of treatment.

## Conclusion

In our case series, we found the prevalence of vertical deviation following HToVR was 54%. None of the patients with an induced postoperative vertical deviation reported diplopia or required further surgery. Interestingly, we have shown that the postoperative BSV was restored as an additional three patients achieved BSV.

## Appendix

**Table:**

ABBREVIATION	MEANING

A	Alternating

R	Right

L	Left

ET	Esotropia

E	Esophoria

XT	Exotropia

X(T)	Intermittent Exotropia

X	Exophoria

HT	Hypertropia

H	Hyperphoria

HypoT	Hypotropia

F/u	Follow up
